# Supplementary material for: Cryo-EM structure of transcription termination factor Rho from Mycobacterium tuberculosis reveals bicyclomycin resistance mechanism
Source: Commun Biol. 2022 Feb 9;5:120. doi: 10.1038/s42003-022-03069-6 (PMC8828861; doi:10.1038/s42003-022-03069-6)
Supplement: Supplementary file 2 — Supplementary information [file 42003_2022_3069_MOESM2_ESM.pdf]

Cryo-EM structure of transcription termination factor Rho from *Mycobacterium tuberculosis* reveals bicyclomycin resistance mechanism

Emmanuel Saridakis, Rishi Vishwakarma, Josephine Lai-Kee-Him, Kevin Martin, Isabelle Simon, Martin Cohen-Gonsaud, Franck Coste, Patrick Bron, Emmanuel Margeat, and Marc Boudvillain

(Supplementary information)

SUPPLEMENTARY METHODS

Crystallization trials with $_{Mtb}$Rho

We performed extensive crystallization trials with the purified WT $_{Mtb}$Rho and T501K mutant. The attempts involved the protein unliganded, as well as complexed with various RNA and DNA oligonucleotides, and ATP analogues[1,2]. None of these trials produced crystals.

We next optimised the oligonucleotide/ cofactor combination using the thermal shift assay method[3,4]. The thermostability (Tm) of the protein was measured in the apo- form and upon mixing with various potential ligands. ADP.BeF3 and ADP.AlF4, combined with oligonucleotides dC_{20}, dC_{34}, and 5'r(UUCCCCCCCC), all led to an increase in thermostability[5] and crystallization screening experiments with most of these combinations were conducted, but still without success.

We thus decided to perform *in situ* random proteolysis, with the addition of trypsin or chymotrypsin directly into crystallisation drops[6,7]. Crystals were obtained with the T501K mutant incubated with 5'r(UUCCCCCCCC)/ ADP.BeF_3 and trypsin (1.5 mg/mL original stock, added at a 1:10000(v/v) dilution to T501K), in 13% PEG 3350, 0.2 M $NaSO_4$ and 0.1 M Bis-Tris propane pH 7.5. These crystals, however, only diffracted to ca. 6 Å at best.

Since a crystal structure could not be determined with such crystals, several of them were pooled together, washed and dissolved for analysis by high-resolution mass spectroscopy (at the MS core facility of CBM). Several T501K proteolytic fragments were identified reproducibly in the dissolved material, none of them likely to contain the NID. We surmised that these fragments may retain both their folding and their mutual interactions. This would explain that they could form crystals at all and, more significantly, that the molecular replacement solution given by the low resolution crystallographic data indicated an expected hexameric conformation. The likely absence of the NID of $_{Mtb}$Rho from the dissolved crystalline material would confirm its deleterious effect for crystallization.

Preliminary characterization of the $_{Mtb}$Rho-ATP-DNA complex by negative stain

We checked the quality and homogeneity of the $_{Mtb}$Rho-ATP-DNA complex sample by negative stain-electron microscopy. Three microliters of the $_{Mtb}$Rho-ATP-DNA complex at 0.05 mg/ml were applied for 2 min on glow-discharged carbon-coated grids and, then, were negatively stained with uranyl acetate 1 % for 1 min. Observation of EM grids was carried out on a JEOL 2200FS FEG Transmission Electron Microscope (TEM) operating at 200 kV under low-dose conditions (total dose of 20 electrons/$Å^2$) in the zero-energy loss mode with a slit width of 20 eV. Images were recorded on a 4K × 4K slow-scan charge-coupled device camera (Gatan Inc.) at a nominal magnification of ×50,000 with defocus ranging from 0.5 to 1.0 µm. In total, 45 micrographs were recorded. The picking of particles was performed using e2boxer from Eman2 package[8] and 2D class averages computed using RELION-3.1.0.

Characterization of the open Rho hexamers

In order to compare the 3D ring structures of $_{Ec}$Rho and $_{Mtb}$Rho, two distance parameters, the ring-opening and rise between protomers at the gap (**Supplementary figure 5**), were defined according to the following protocol. For each open ring structure of $_{Mtb}$Rho (this work) and $_{Ec}$Rho in isolation 3 (PDB 1PVO, 1PV4, 1XPO, 6WA8) or in complex with RNAP (PDB 6XAS, 6Z9P, 7ADB), the MatchMaker module of Chimera was used to superimpose the C-terminal domain of the protomer 1 (residues 1-129 for $_{Ec}$Rho and 1-306 for $_{Mtb}$Rho were omitted) on protomer 1 of the reference, i.e. closed $_{Ec}$Rho ring structure (PDB 3ICE) (see **Supplementary figure 7** for protomer numbering). Then, the center of mass (COM) of the C-terminal domain of each Rho protomer was calculated using the CALCOM server (http://bioinformatica.isa.cnr.it/CALCOM) and displayed as a 2 Å radius sphere (**Supplementary figure 7**). The ring opening parameter represents the distance in Angstroms between the COMs of protomers

1 and 6 at the gap (**Supplementary figure 7,** dash line) minus the distance between COMs 1 and 6 in the 3ICE reference. The rise parameter is defined as the shortest distance between the COM of protomer 6 of the structure of interest and a plane passing through the six COMs of the 3ICE reference.

Supplementary table 1: Nucleic acid sequences

DNA template (top strand)<br>*Termination assay*<br>*(promoter sequence underlined)*	5’d(TTATCAAAAAGAGTATTGACTTAAAGTCTAACCTATAGGATACTTACAGCCATGT<br>AGTAAGGAGGTTGTATGGAACAACGCATAACCCTGAAAGATTATGCAATGCGCTTT<br>GGGCAAACCAAGACAGCTAAAGATCTCGGCGTATATCAAAGCGCGATCAACAAGG<br>CCATTCATGCAGGCCGAAAGATTTTTTTAACTATAAACGCTGATGGAAGCGTTTAT<br>GCGGAAGAGGTAAAGCCCTTCCCGAGTAACAAAAAAACAACAGCATAAATAACCC<br>CGCTCTTACACATTCCAGCCCTGAAAAAGGGCATCAAATTAAACCACACCTATGGT<br>GTATGCATTTATTTGCATACATTCAATCAATTGTTATCTAAGGAAATACTTACATATG<br>GTTCGTGCAAACAAACGCAACGAGGCTCTACGAATCGAGAGTGCGGGTAATACTC<br>AGCCAGCTTTGTCATGG)
RNA strand<br>*Duplex unwinding assay*	5’r(GGACUUCUCCUCUGUCUCCUUCUUCCUUCUCCUCUGUCUCCUUCUUCCUCG<br>ACCUAUUGAGUUUGAAUUUAUCGAUGGUAUCAGAUCUGGAUCCUCGAGAAGCU<br>GCGGGUACCGAGCUCGAAUUCAUCG)
DNA strand<br>*Duplex unwinding assay*	5’d(CGATGAATTCGAGCTCGGTACCCGCAGCTTCTCGAGGATCCAGATCTGATACC<br>ATCG)
Trap oligo<br>*Duplex unwinding assay*	5’d(CGATGGTATCAGATCTGGATCCTCGAGAAGCTGCGGGTACCGAGCTCGAATT<br>CATCG)

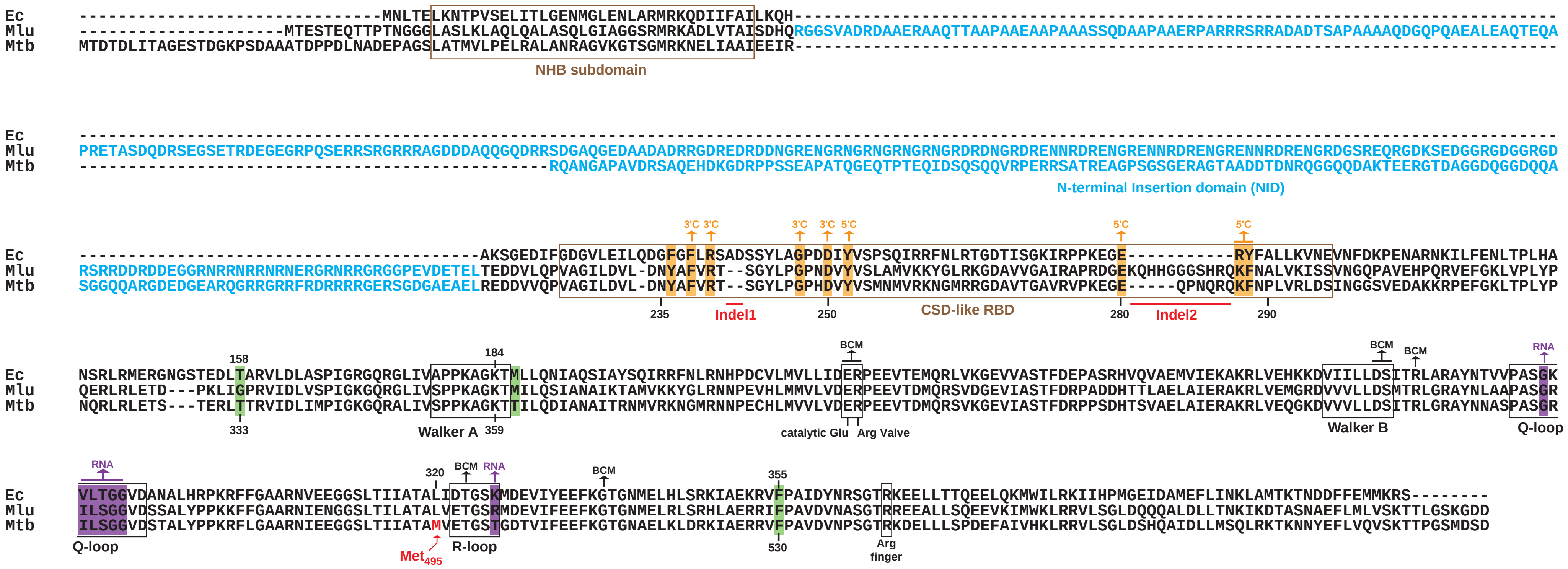

Supplementary Figure 1: Alignment of the Rho sequences from *E. coli* (Ec), *M. luteus* (Mlu), and *M. tuberculosis* (Mtb). Positions corresponding to PBS and SBS residues in $_{Ec}$Rho are boxed in orange and purple, respectively. Residues contacting the base moiety of ATP are boxed in green.

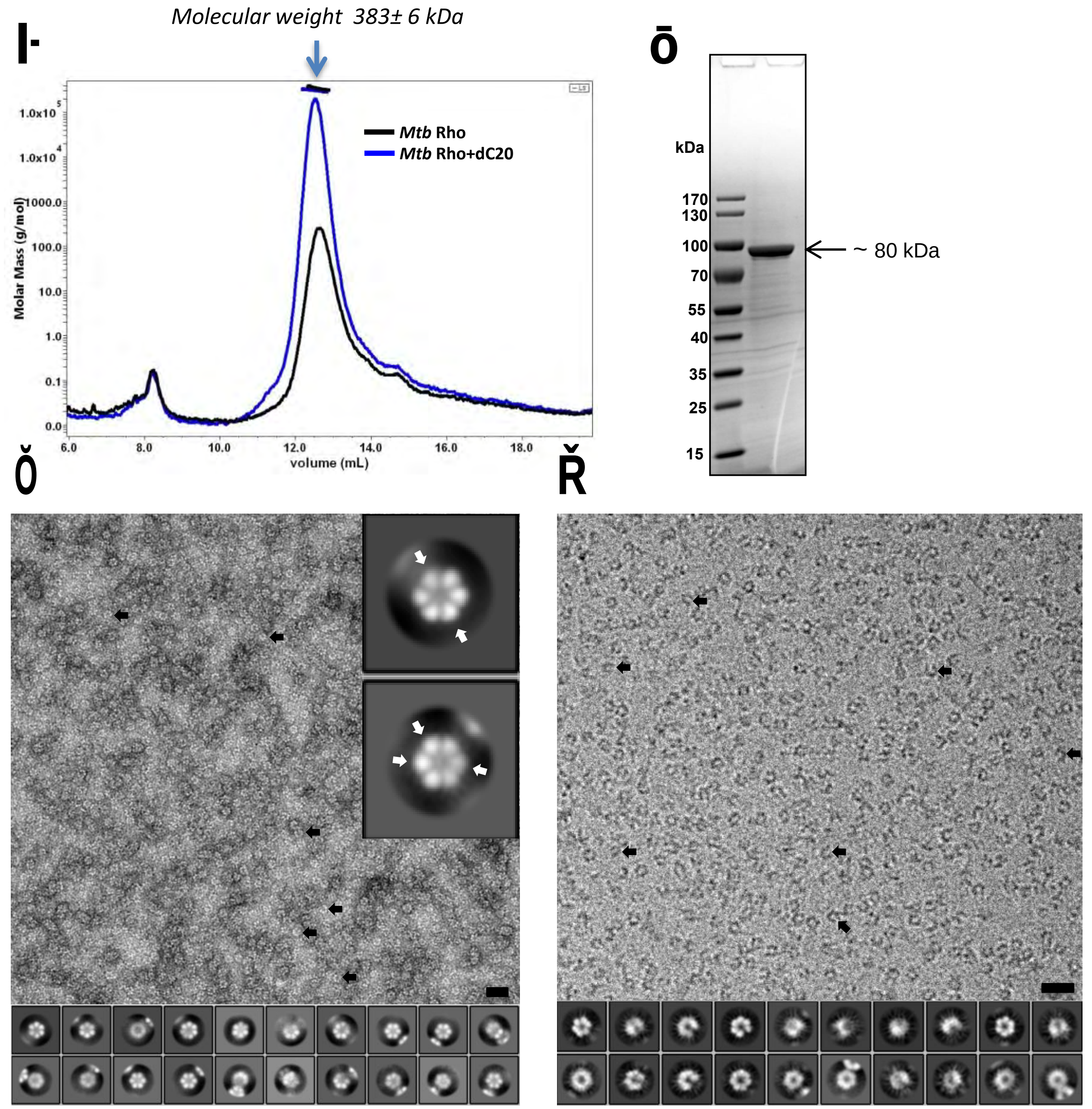

Supplementary Figure 2 : Purification and preliminary characterization of $_{Mtb}$Rho. **(I·)** Size Exclusion Chomatography & Multi- Angle Light Scattering profile. The highest peak (12-14 mL) corresponds to a molecular mass (MM) of 383± 6 kDa, close to the theoretical MM of $_{Mtb}$Rho hexamer (6 x 65 kDa). **(ō)** SDS-PAGE of the highest peak fraction shows a unique band at ~80 kDa. The difference between this apparent MM and the theoretical MM of $_{Mtb}$Rho is due to an abnormally slow migration, as reported before (27). **(Ŏ)** Negative stain image of $_{Mtb}$Rho with Mg-ATP and dC20 (in-house JEOL2200FS microscope) and corresponding 2D class averages. $_{Mtb}$Rho particles are homogeneous, showing and confirming the hexameric organization of $_{Mtb}$Rho, where only top views are visible when $_{Mtb}$Rho particles are bound to the carbon film. 2D class averages mainly show a closed ring-like organization even if open ring-like particles can also be observed (black arrows). The zoomed-in views of the two best 2D class averages reveal that the intensity of protomers is not uniform, as outlined with white arrows. **(Ř)** Cryo-EM image of $_{Mtb}$Rho in the presence of Mg-ATP and dC$_{20}$ (in-house JEOL2200FS microscope) and corresponding 2D class averages. Image and 2D class averages of frozen-hydrated $_{Mtb}$Rho complexes reveal various orientations of particles in ice where the vast majority of particles display an open conformation, as indicated by black arrows. So $_{Mtb}$Rho is suitable for high resolution acquisition. Scale bars: 60 nm

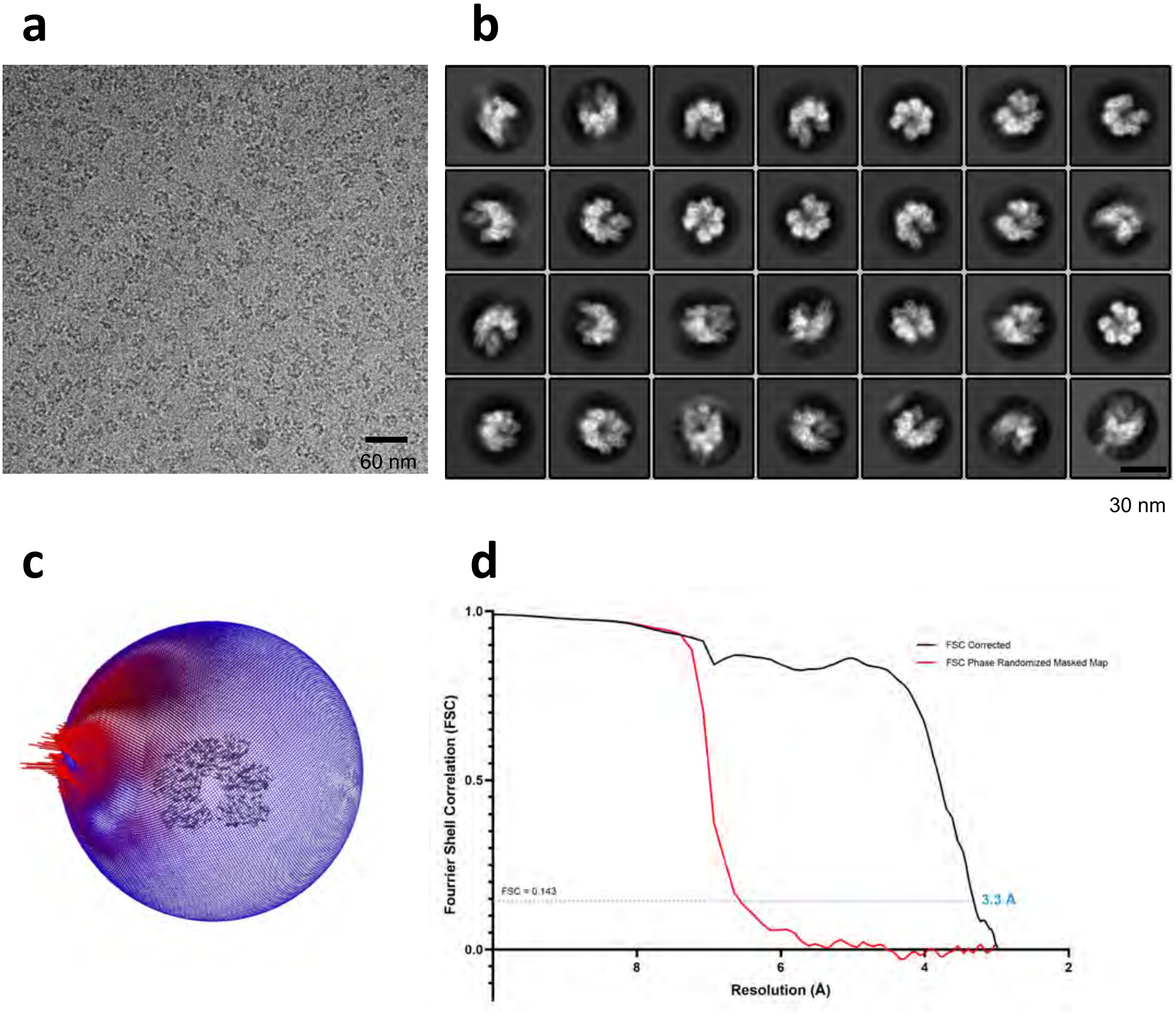

Supplementary Figure 3 : Cryo-EM analysis of $_{Mtb}$Rho transcription factor complex. **(a)** Representative motion-corrected electron micrograph embedded in vitreous ice (0.81 Å per pixel, 1.86 um defocus) of the $_{Mtb}$Rho dataset (scale bar, 60 nm). This image has been lowpass-filtered to 1 nm for better visibility. **(b)** Representative reference-free two-dimensional class averages of $_{Mtb}$Rho complex (scale bar, 30 nm), from Relion two-dimensional classification. These 2D class averages show secondary structure elements. **(c)** Euler angle distribution plots of $_{Mtb}$Rho particles, reflecting the initial angular distribution. The number of particles with respective orientations are represented by length and colored cylinders, ranging from blue to red. **(d)** High resolution refinement : the Fourier Shell Correlation (FSC) curves between independently refined half-maps at the final stage of the processing indicates an average resolution of 3.3 Å according to the FSC=0.143 criterion.

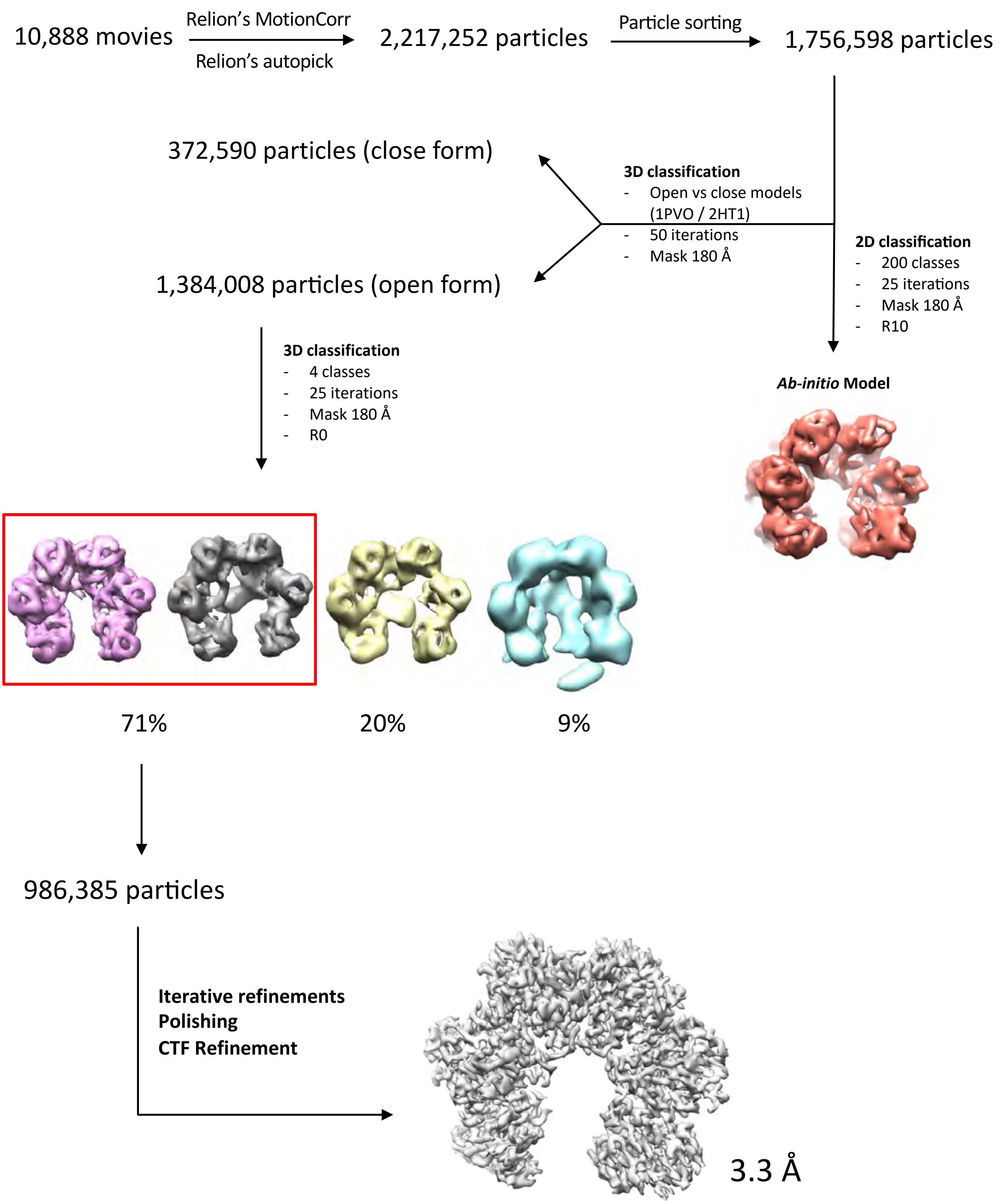

Supplementary Figure 4: Flowchart of the overall data processing pipeline in Relion. See methods for details.

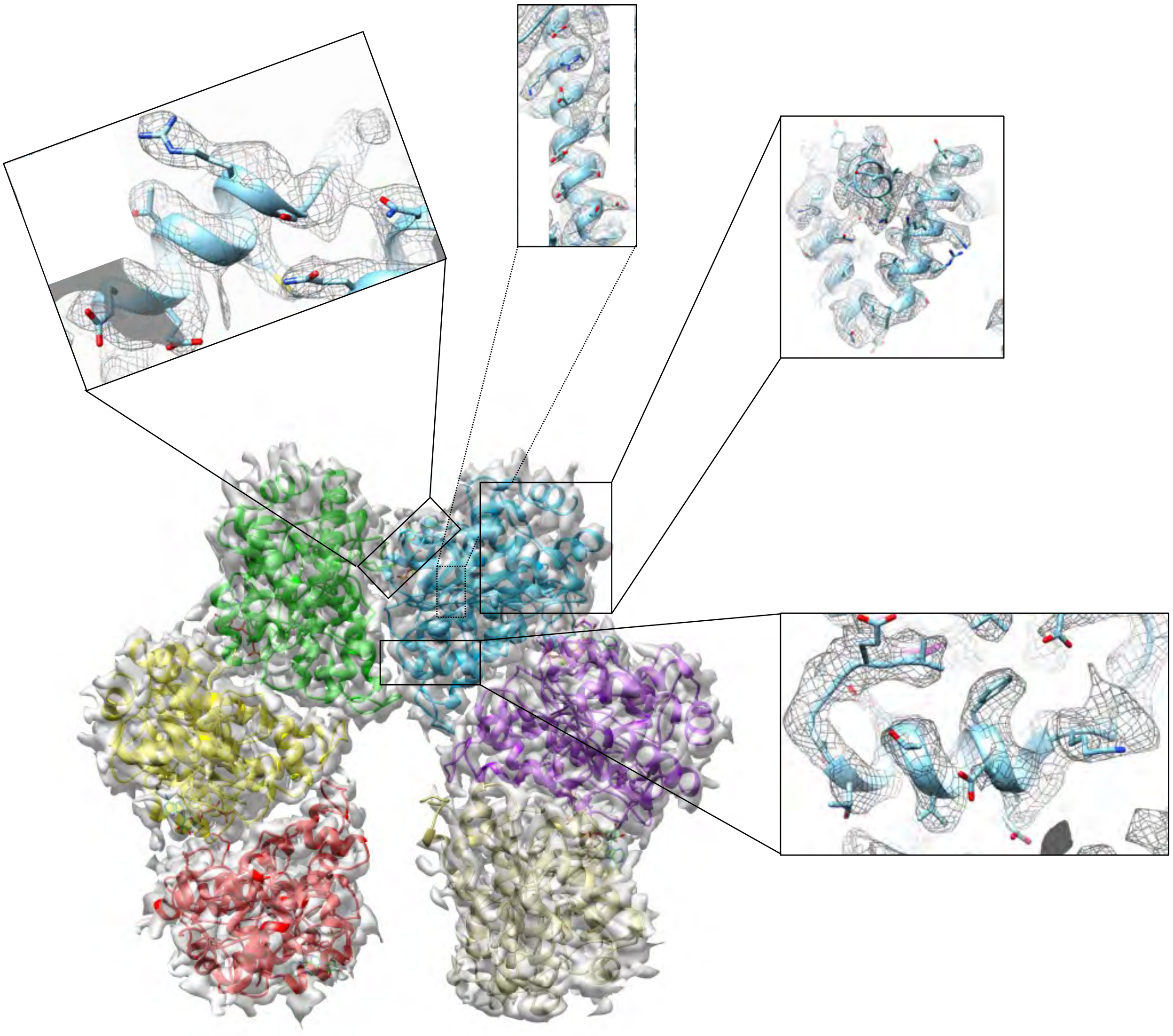

Supplementary Figure 5: Representative model and density features (chain D) fitted into the 3.3A Å cryo-EM-based atomic model.

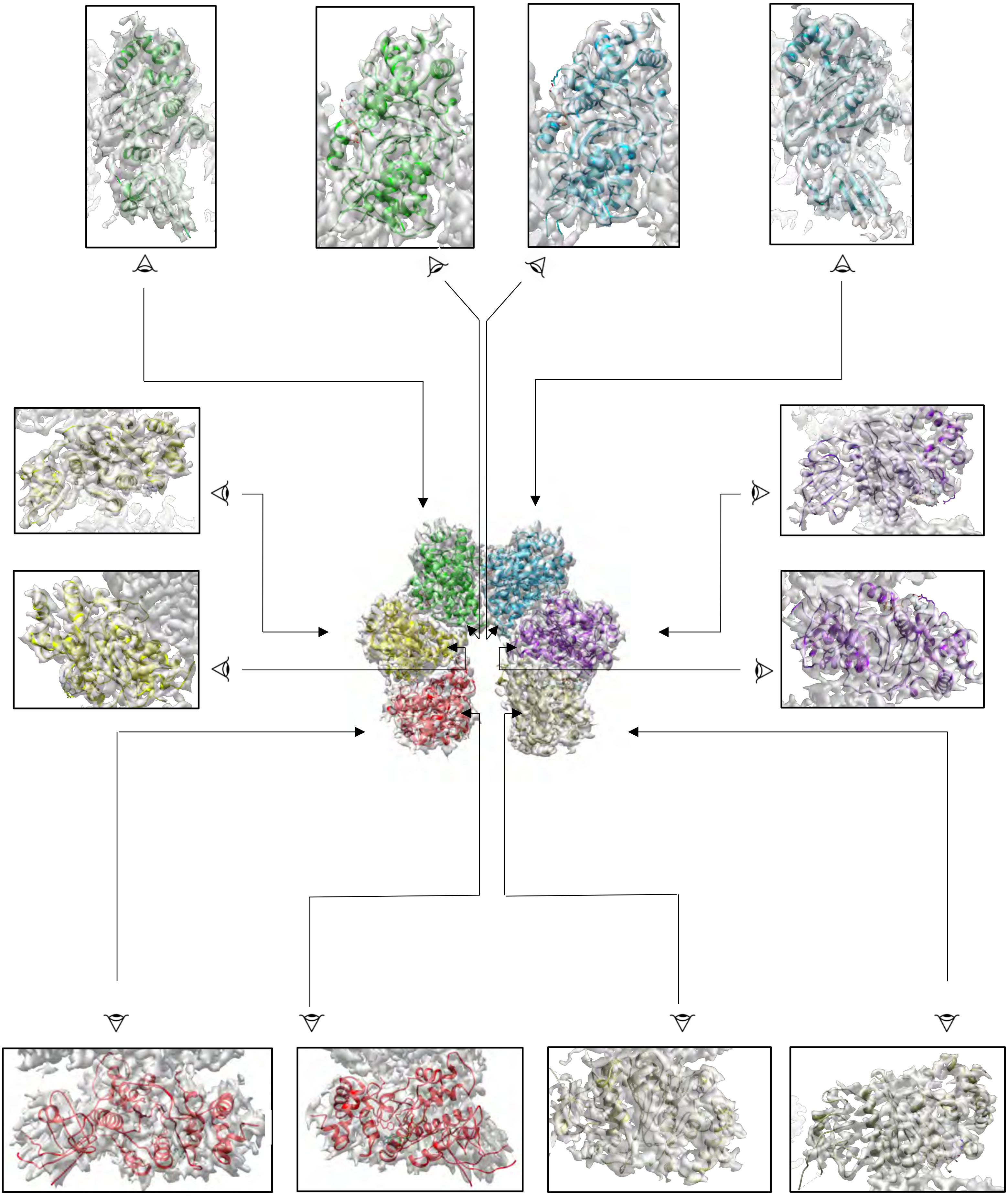

Supplementary Figure 6: Representative views (inside and outside) of each $_{Mtb}$Rho protomer.

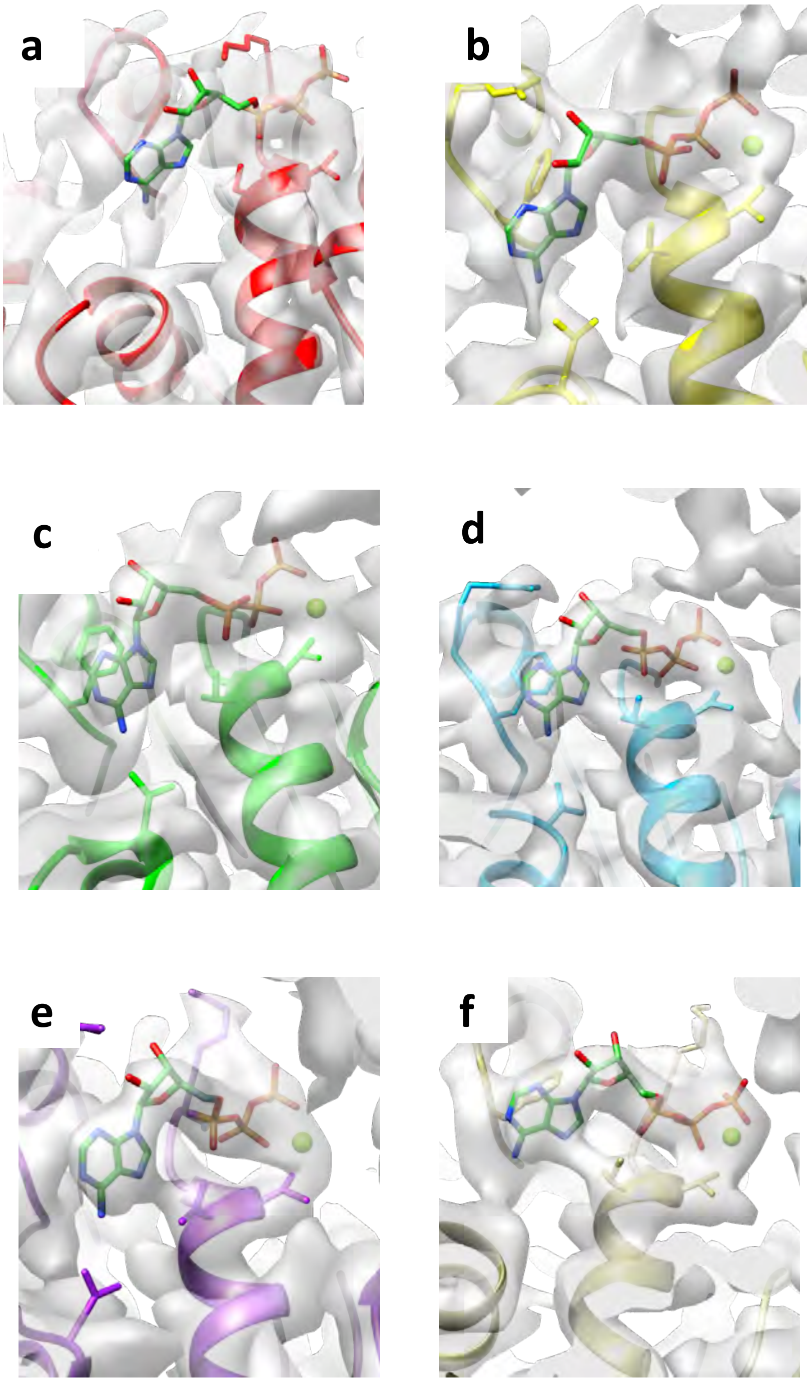

Supplementary figure 7. ATP binding sites fitted into the cryo-EM map**.** All ATP binding sites, from chain A (red, **a**), B (yellow, **b**), C (green, **c**), D (cyan, **d**), E (purple, **e**) and F (kaki, **f**) of $_{Mtb}$Rho.

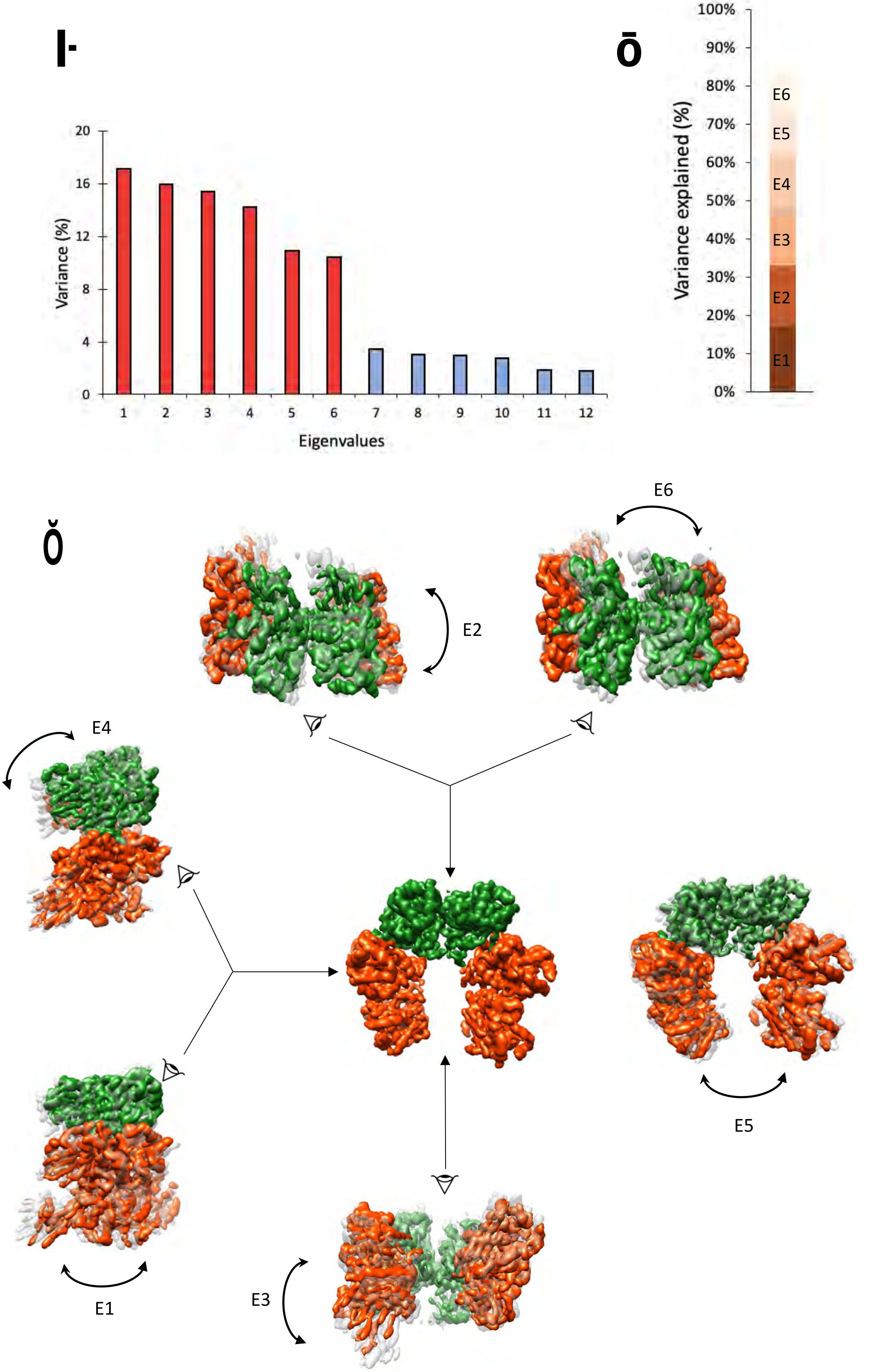

Supplementary Figure 8 : Structural flexibility and dynamics of the $_{Mtb}$Rho hexamer. We observe interdomain and intersubunit movements in the structure. **(a)** Contribution of each of 12 eigenvectors to the variance of the final cryo-EM map. **(b)** Eigenvectors 1 to 6 correspond to most (83%) of the variance. **(c)** The $_{Mtb}$Rho consensus map (3.4 Å) used for multibody 3D-refinement is shown in the middle, with two bodies colored interdomain dynamics (in gray). The most resolved domains are in green (chains C/D) while others are in orange/red. Maps corresponding to the first 6 eigenvectors are shown. E4 is part of E1 while E6 is part of E2.

.

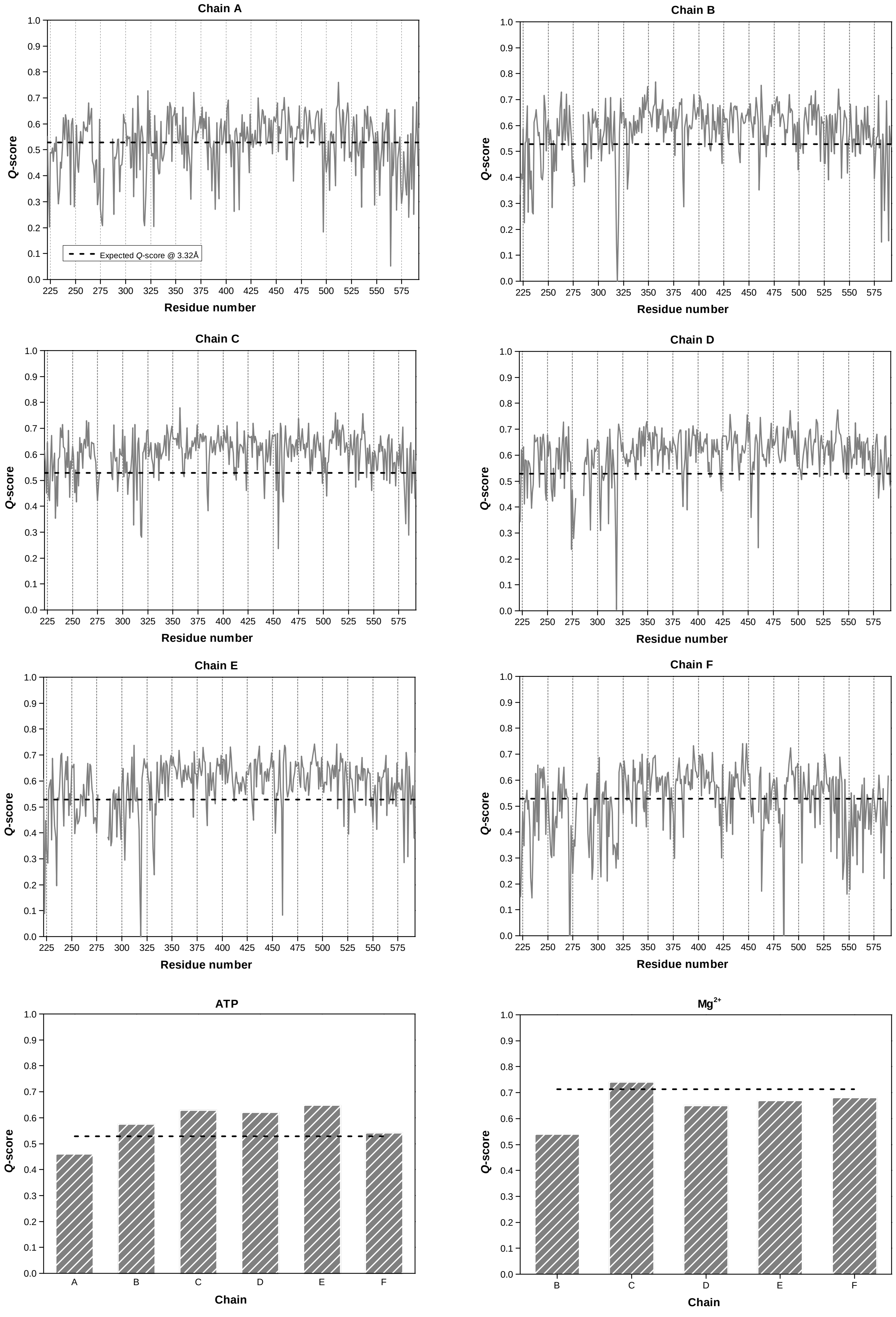

Supplementary Figure 9: Residue resolvability in cryo-EM map. Q scores were calculated with the MapQ plugin of UCSP Chimera. Dashed lines represent the average Q-score expected at 3.32 Å resolution.

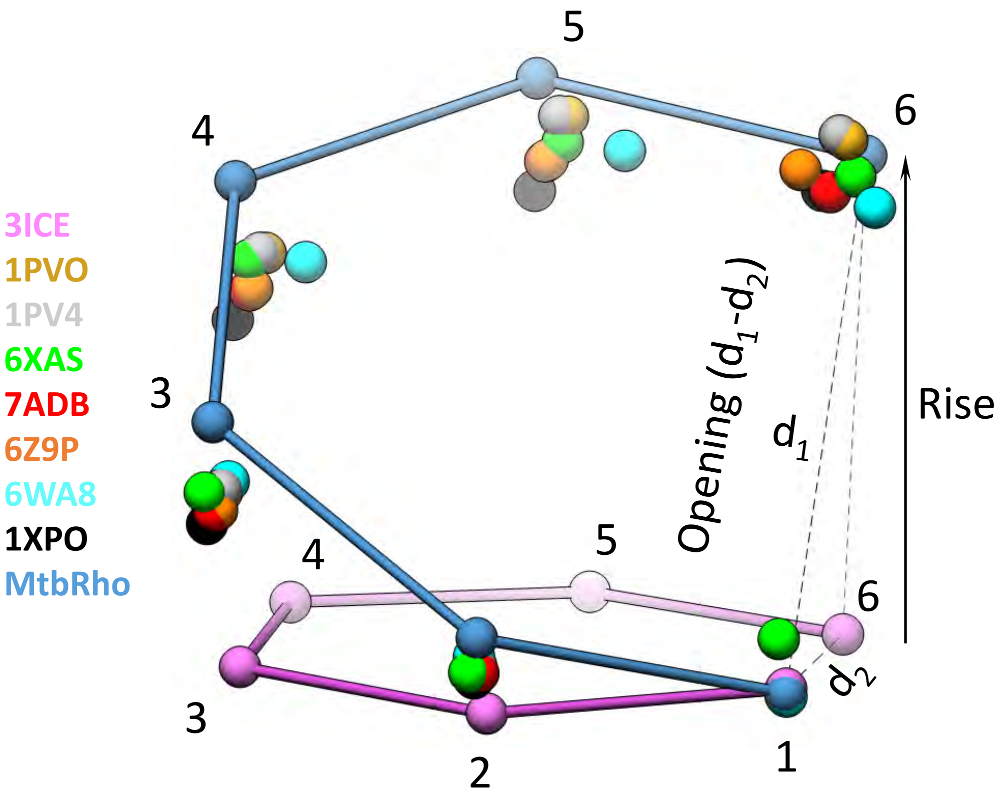

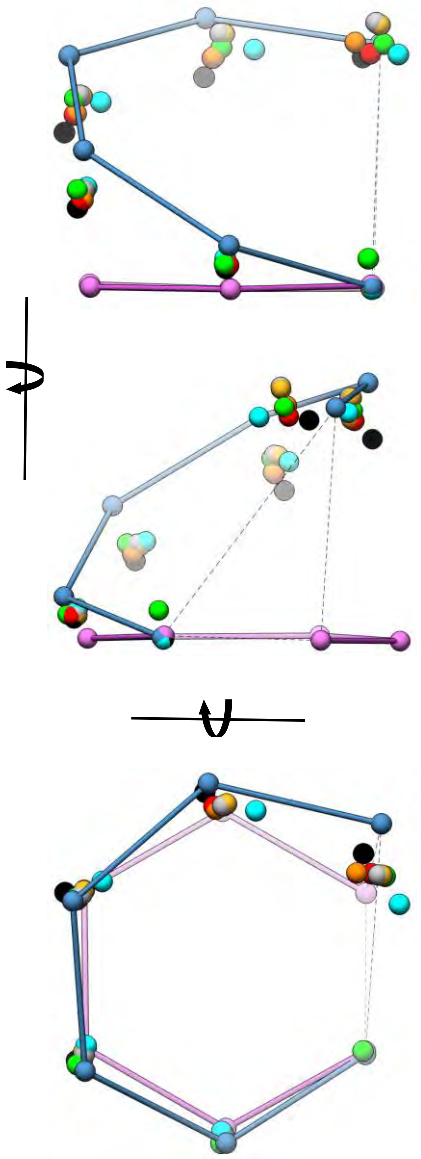

	PDB#	Opening (Å)	Rise (Å)
$_{Ec}$Rho (X-ray)	3ICE	0	0
	1PVO	25	50
	1PV4	24	51
	1XPO	22	45
$_{Ec}$[Rho:RNAP] complexes (cryoEM)	6XAS	17	43
	7ADB	20	46
	6Z9P	22	49
	6WA8	18	45
$_{Mtb}$Rho (this work)	7OQH	27	47

Supplementary Figure 10: Rho ring parameters.

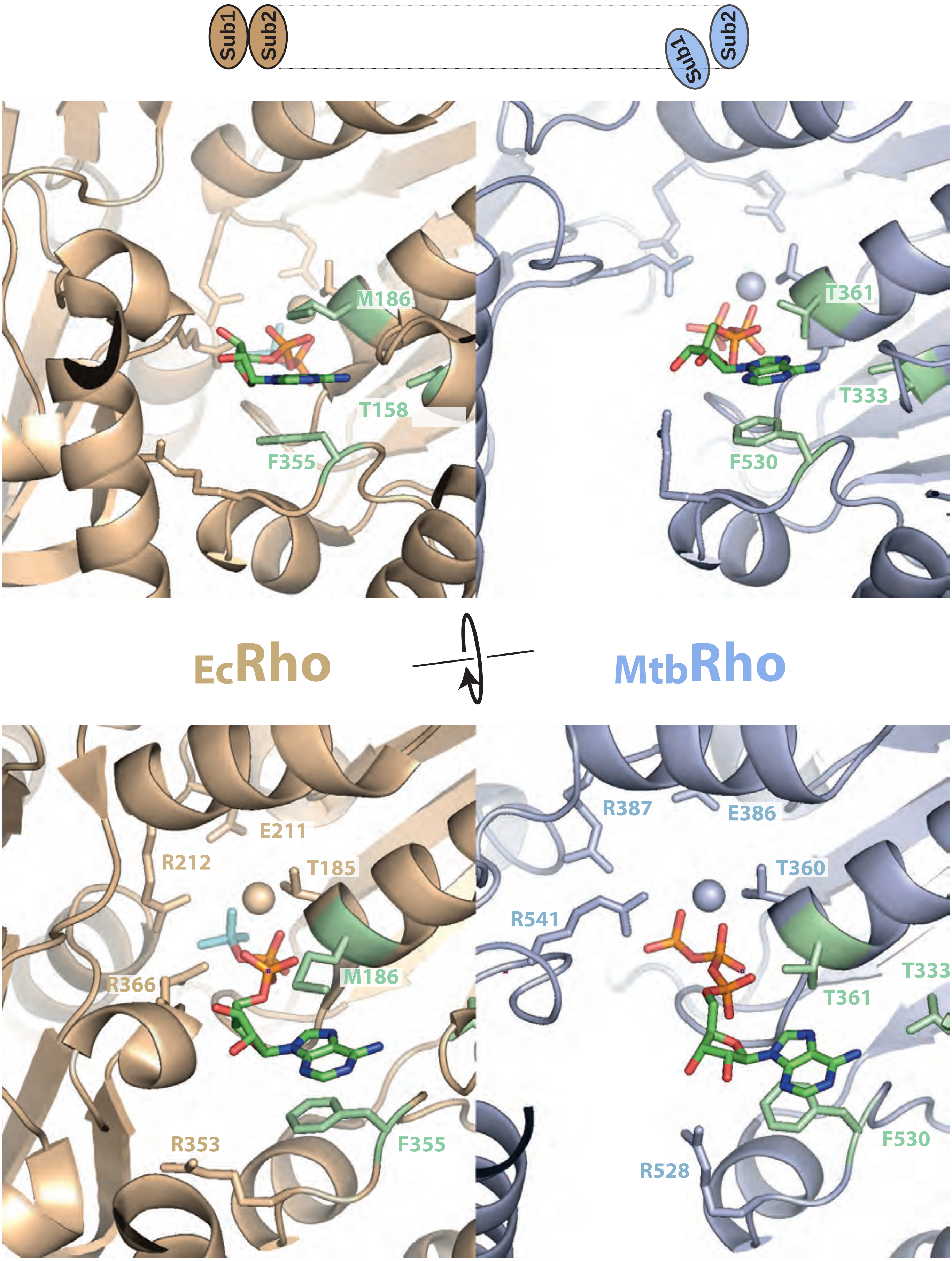

Supplementary Figure 11: Organization of the ATPase pockets in the closed $_{Ec}$Rho hexamer (PDB 5JJI) and open $_{Mtb}$Rho hexamer (this work). The B/C subunit interface of $_{Ec}$Rho (interface in a productive ATPase conformation in the assymetric closed hexamer) and C/D subunit interface of $_{Mtb}$Rho (best resolved interface) were used for comparison. The BeF_3 ion in the $_{Ec}$Rho structure is represented by cyan sticks. Mg^{2+} ions are shown as spheres. The respective dispositions of subunits at the interfaces are schematically depicted above figures.

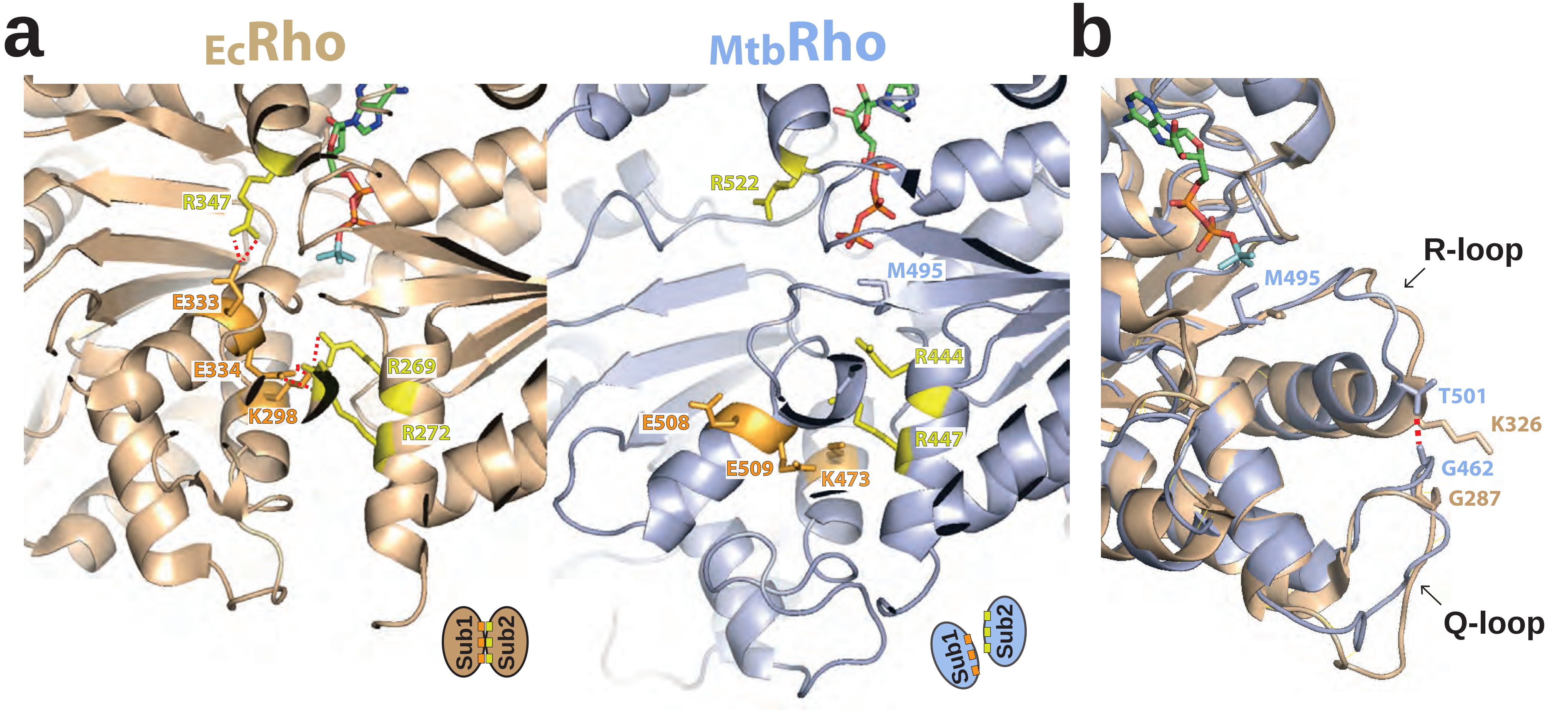

Supplementary Figure 12: Organization of the allosteric communication network in the closed $_{Ec}$Rho hexamer (PDB 5JJI) and open $_{Mtb}$Rho hexamer (this work). **(a)** Network contacts between subunits are disrupted in $_{Mtb}$Rho, which is consistent with an unproductive hexamer conformation. **(b)** The Thr501 side-chain of $_{Mtb}$Rho mediates an interaction between the Q- and R-loops. In $_{Ec}$Rho, the corresponding Lys326 side-chain lies in the central channel where it can contact and translocate the RNA chain[1].

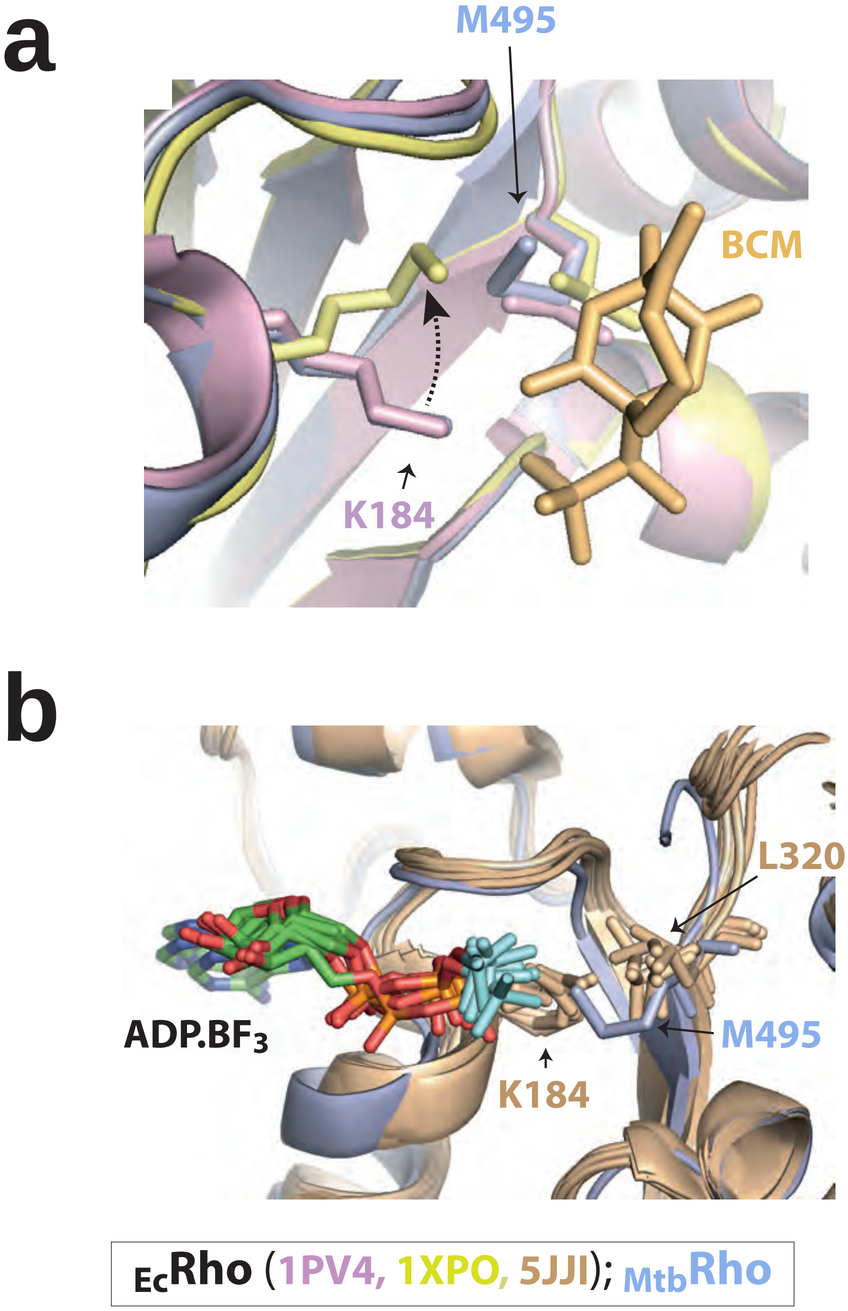

Supplementary Figure 13: The bulky $_{\mathrm{Mtb}}$Met495 side-chain lies in the path of a mobile lysine from the ATPase Walker A motif ($_{\mathrm{Mtb}}$Lys380, i.e. $_{\mathrm{Ec}}$Lys184 in $_{\mathrm{Ec}}$Rho). **(a)** The $_{\mathrm{Ec}}$Lys184 side-chain adopts distinct conformations in BCM-free (in pink) and BCM-bound (in light yellow) $_{\mathrm{Ec}}$Rho (protomers C from PDB 1PV4 and 1XP0, respectively). The $_{\mathrm{Mtb}}$Rho protomer C is in blue and BCM in light orange. **(b)** Motion of the $_{\mathrm{Ec}}$Lys184 side-chain in the assymetric, closed $_{\mathrm{Ec}}$Rho hexamer (PDB 5IJJ) as a function of the chemical/conformational state of the ATPase pocket. The six $_{\mathrm{Ec}}$Rho protomers (in light brown) have been aligned with $_{\mathrm{Mtb}}$Rho protomer C (in blue). The BeF_3 ion is shown in cyan sticks.

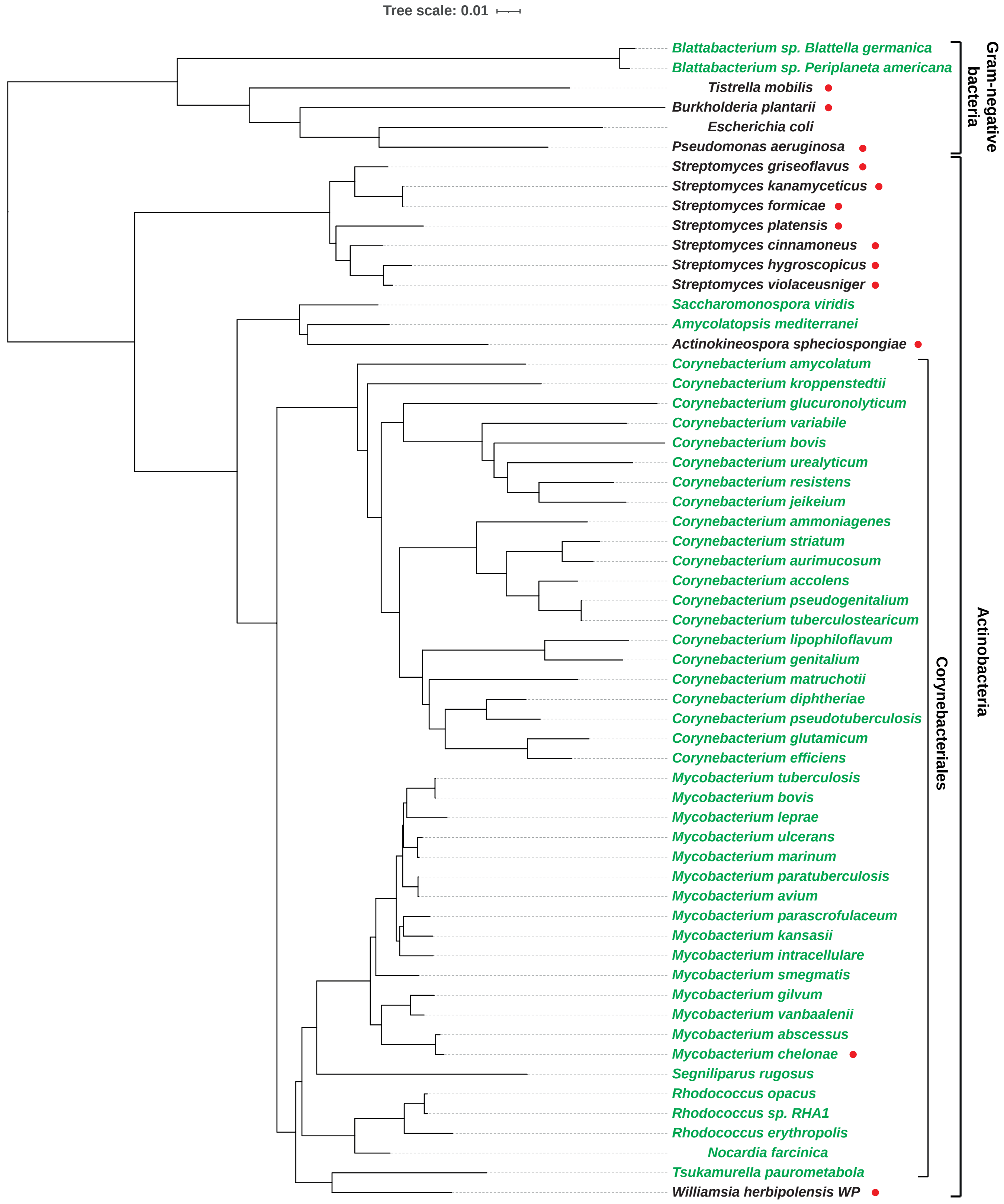

Supplementary Figure 14: Phylogenetic distribution of the Leu→Met substitution in the BCM-binding pocket of Rho. Species bearing the Leu→Met mutation are in green. Most belong to the Corynebacteriales order. *Blattabacterium sp.* (Bacteroidetes phylum) are insect endosymbionts. Species bearing the cluster of genes for BCM biosynthesis are identified by a red dot. The unrooted tree has been built with the Seaview 4.6.4 software.

SUPPLEMENTARY REFERENCES

1. Thomsen, N.D. & Berger, J.M. Running in reverse: the structural basis for translocation polarity in hexameric helicases. *Cell* **139**, 523-34 (2009).

2. Thomsen, N.D. & Berger, J.M. Crystallization and X-ray structure determination of an RNA-dependent hexameric helicase. *Methods Enzymol* **511**, 171-90 (2012).

3. Pantoliano, M.W. et al. High-density miniaturized thermal shift assays as a general strategy for drug discovery. *J Biomol Screen* **6**, 429-40 (2001).

4. Vedadi, M. et al. Chemical screening methods to identify ligands that promote protein stability, protein crystallization, and structure determination. *Proc Natl Acad Sci U S A* **103**, 15835-40 (2006).

5. Saridakis, E. & Coste, F. Thermal Shift Assay for Characterizing the Stability of RNA Helicases and Their Interaction with Ligands. *Methods Mol Biol* **2209**, 73-85 (2021).

6. Dong, A. et al. In situ proteolysis for protein crystallization and structure determination. *Nat Methods* **4**, 1019-21 (2007).

7. Wernimont, A. & Edwards, A. In situ proteolysis to generate crystals for structure determination: an update. *PLoS One* **4**, e5094 (2009).

8. Tang, G. et al. EMAN2: an extensible image processing suite for electron microscopy. *J Struct Biol* **157**, 38-46 (2007).
