# Supplementary material for: Cryo-EM structure of transcription termination factor Rho from Mycobacterium tuberculosis reveals bicyclomycin resistance mechanism
Source: Commun Biol. 2022 Feb 9;5:120. doi: 10.1038/s42003-022-03069-6 (PMC8828861; doi:10.1038/s42003-022-03069-6)
Supplement: Supplementary file 3 — Description of Additional Supplementary Files [file 42003_2022_3069_MOESM3_ESM.pdf]

Description of Additional Supplementary Files

File name: Supplementary Data 1
Description: Uncropped gel scans.
